# The Effects of Early Nutritional Intervention on Oral Mucositis and Nutritional Status of Patients With Head and Neck Cancer Treated With Radiotherapy

**DOI:** 10.3389/fonc.2020.595632

**Published:** 2021-02-01

**Authors:** Zhuangzhuang Zheng, Xin Zhao, Qin Zhao, Yuyu Zhang, Shiyu Liu, Zijing Liu, Lingbin Meng, Ying Xin, Xin Jiang

**Affiliations:** ^1^ Department of Radiation Oncology, The First Hospital of Jilin University, Changchun, China; ^2^ Jilin Provincial Key Laboratory of Radiation Oncology & Therapy, The First Hospital of Jilin University, Changchun, China; ^3^ NHC Key Laboratory of Radiobiology, School of Public Health, Jilin University, Changchun, China; ^4^ Department of Orthopedic, The Second Hospital of Jilin University, Changchun, China; ^5^ Department of Hematology and Medical Oncology, Moffitt Cancer Center, Tampa, FL, United States; ^6^ Key Laboratory of Pathobiology, Ministry of Education, Jilin University, Changchun, China

**Keywords:** early nutritional intervention, head and neck cancer, radiotherapy, oral mucositis, malnutrition

## Abstract

Radiation-induced oral mucositis (RIOM) is a common side effect after radiotherapy (RT) in head and neck cancer (HNC) patients. RIOM patients with severe pain have difficulty in eating, which increases the incidence of malnutrition and affects patients’ quality of life and the process of RT. The mechanism of RIOM is not fully understood, and inflammatory response and oxidative stress appear to be important for RIOM occurrence and development. The nutritional status of patients is very important for their RT tolerance and recovery. Malnutrition, which can lead to anemia, low protein, decreased immunity and other problems, is an important clinical factor affecting tumor progression and treatment. Recent studies have shown that early nutritional intervention can ameliorate oral mucositis and nutritional status of patients with HNC. However, in clinical practice, early nutritional intervention for patients with HNC is not a conventional intervention strategy. Therefore, this review summarized the possible pathogenesis of RIOM, commonly used assessment tools for malnutrition in patients, and recent studies on the effects of early nutritional interventions on RIOM and nutritional status of patients with HNC. We hope to provide the basis and reference for the clinical application of early nutritional intervention models.

## Introduction

Head and neck cancer (HNC) is the sixth most common cancer worldwide with more than half a million new cases diagnosed each year (1, 2). Because of the limitations imposed by the complicated anatomical structure of the head and neck to the operation, radiotherapy (RT) has become the main treatment method for HNC. However, damage to the normal tissue surrounding the tumor is inevitable. The most common manifestations of injury caused by RT are mucositis, dysphagia, pharyngeal pain, taste disorders, mouth dryness, nausea, vomiting, and anorexia, which may adversely affect the nutritional status of patients and lead to a decline in their quality of life (3–6).

Radiation-induced oral mucositis (RIOM) is a common side effect of RT for HNC. Studies have shown that patients with HNC have oral mucositis at various degrees, when the radiation dose reaches a certain level, and the incidence of ≥ Grade 3 mucositis is as high as 56% (7, 8). RIOM, which usually starts at around the 5^th^ to 10^th^ RT fraction, occurs in > 80% of patients during RT. Hyperemia, erythema, and erosion may occur in patients’ mucosa until severe ulcers and fibrosis appear (9). Severe pain makes it difficult for patients to eat and leads to malnutrition, which affects patients’ quality of life and the course of RT (10, 11).

Malnutrition, which is caused by failure of food intake to provide the required energy, is an important clinical factor in the progression and treatment of cancer (12). RIOM is one of the key barriers to food intake. Most HNC patients experience weight loss after RT, and many suffer from moderate to severe malnutrition, which affects patients’ quality of life and treatment process (13, 14). Weight loss in patients leads to changes in body shape, decreases immobilization of head and neck masks, and affects RT accuracy. Malnutrition can also lead to anemia, low protein, decreased immunity, and increased complications during RT.

Nutritional intervention is important for maintaining the nutritional status of patients with HNC. International guidelines recommend strengthening nutritional consultation and oral nutritional supplement as nutritional interventions for patients with HNC undergoing chemoradiotherapy (15, 16). Nutritional interventions that emphasize protein targets during RT may reduce severity of oral mucositis. Studies have shown that oral mucositis is less severe when patients with HNC achieve the corresponding protein and calorie intake targets during RT (17–19). However, in clinical practice, nutritional intervention usually begins when patients develop oral mucositis or severe gastrointestinal reactions that result in restricted feeding. The effect is limited, and it is difficult to effectively improve the nutritional status of patients. Therefore, early nutritional intervention may become an important treatment to prevent malnutrition. However, currently, early nutritional intervention for patients with HNC is not a conventional nutritional intervention strategy.

The purpose of this review was to analyze the effects of nutritional intervention on radiation-induced oral mucositis and malnutrition in patients in different periods, hence to provide a basis and reference for the application of early nutritional intervention strategies in clinical practice. Therefore, we summarized and compared recent literature on the effects of traditional nutritional intervention and early nutritional intervention on the therapeutic outcome, oral mucosal complications, quality of life, and nutritional status of patients with HNC.

## Oral Mucositis and Malnutrition in HNC Patients With RT

### Radiation-Induced Oral Mucositis

RIOM is one of the most important toxic reactions of normal tissue during RT for in HNC patients (8, 20). RIOM is classified into five grades according to Radiation Therapy Oncology Group standards (21): No changes were observed in Grade 0 mucositis. Grade 1 includes mucositis that causes mild pain or congestion that does not require analgesics. Grade 2 includes the development of patchy mucositis, the requirement for analgesics, or the production of serosanguineous discharge. Grade 3 includes the development of confluent mucositis or severe pain requiring narcotic analgesics, and Grade 4 involves the development of ulcer, necrosis, and bleeding from the area. Severe RIOM can cause severe pain, especially ulcers near the pharynx, which can cause severe pain when swallowed and loss of appetite. In addition, injury to the parotid gland and taste buds leads to decreased saliva and loss of taste, which also seriously affects the patient’s appetite. When fibrosis occurs, the patient has difficulty opening his or her mouth, which can also affect food intake.

The development of RIOM is divided into five stages comprising initiation, signaling, amplification, ulceration, and healing. At the initial stage of injury, radiation deposits energy on biological macromolecules including protein molecules. Furthermore, the epithelium, blood vessels, and mesenchymal cells of the mucosa at the site of injury release reactive oxygen species (ROS) causing damage to DNA. Signal is transduced through matrix metalloproteinases (MMPs), nuclear factor kappa-B (NF-κB), and ceramide pathways in macrophages. The signal amplification stage is mediated by pro-inflammatory cytokines including tumor necrosis factor alpha (TNF-α), interleukin 1β (IL-1β), and IL-6. Subsequently, mucosa desquamation of the epithelium occurs, and the basement membrane is damaged. The oral mucosa loses its protective barrier and eventually ulcerates. At the beginning of the healing phase, basal epithelial cells migrate, proliferate, and differentiate. The ulcer eventually heals. Changes in late stage ulcer are associated with a variety of factors, which may lead to secondary gram-negative bacterial infection. Infection may lead to blocked blood flow and ischemic necrosis, which cause more severe inflammatory changes that eventually heal in the form of fibrosis (20, 22, 23). Patients eat less because of severe oral pain, thereby becoming malnourished. In turn, malnutrition affects the severity and healing time of mucositis and ulcers. In addition, although RT combined with chemotherapy increases local tumor control, it also increases the incidence of ulcerative mucositis and results in interruption of RT (24).

**Figure 1:**
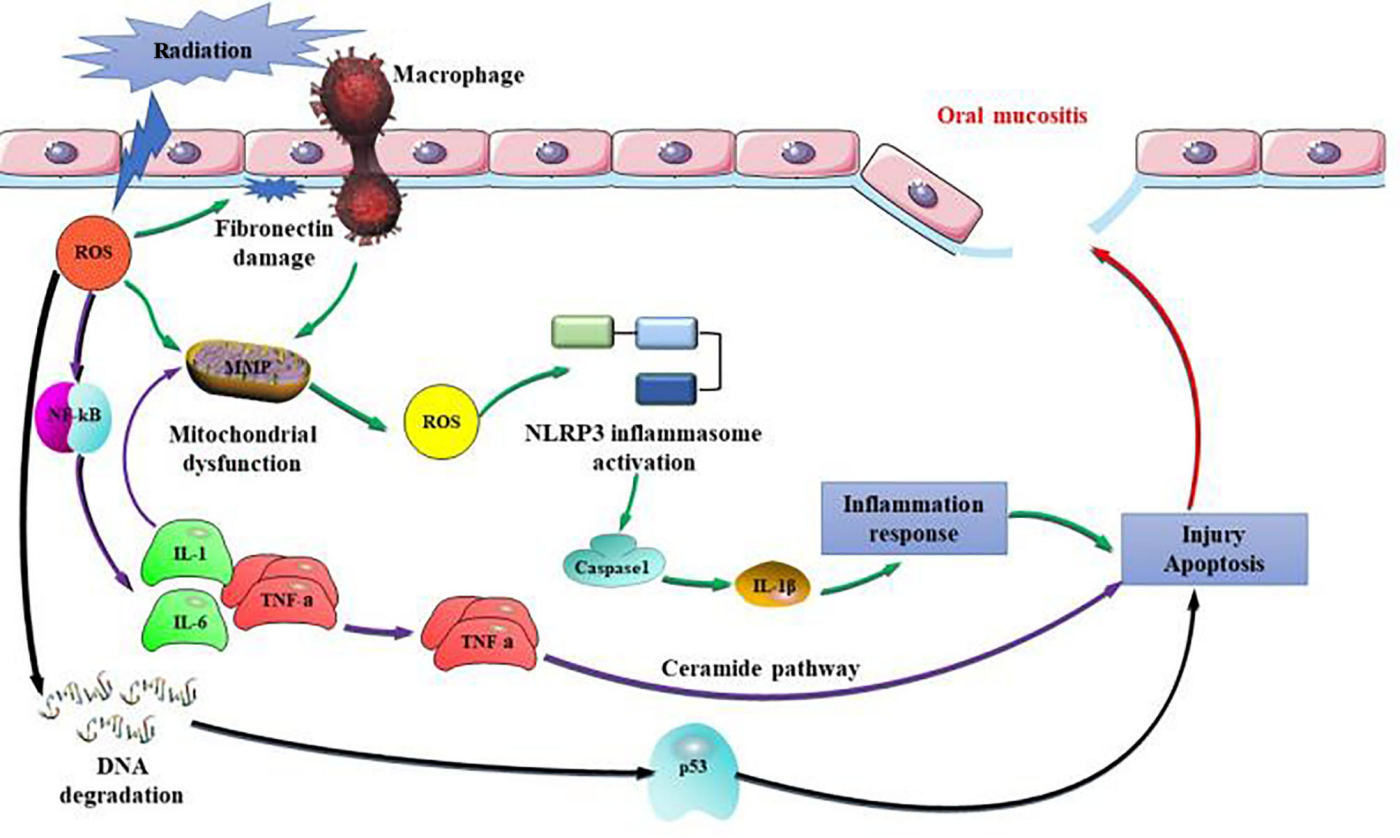
Pathogenic mechanisms of radiation-induced oral mucositis (RIOM). Amplification of reactive oxygen species (ROS) and inflammatory cytokines occurs mainly through the following three steps: (1) activating the nuclear factor kappa-B (NF-κB) pro-inflammatory pathway, stimulating target gene expression, and producing a large number of inflammatory cytokines, such as tumor necrosis factor alpha (TNF-α), interleukin 1 (IL-1), and IL-6, which activate the ceramide pathway, thereby producing large amounts of sphingomyelinase and ceramide synthase and eventually causing more tissue damage and cell apoptosis; (2) fibronectin breakdown, which stimulates macrophages leading to activation of matrix metalloproteinases (MMPs); (3) impaired mitochondria produce more ROS, which activates NOD-like receptor pyrin domain-containing protein 3 (NLRP3) inflammasome pathway. NLRP3 activates caspase-1, which produces IL-1β and leads to apoptosis.

A number of risk factors have been associated with the development of RIOM. These mainly include combined chemotherapy, oral hygiene, low nutrition, early non-use of antibiotics, and smoking (25). Dose is the most important factor affecting RIOM and the dose of oral mucosa is not recommended to exceed 45Gy. With the accumulation of dose, severe RIOM occurred more frequently in weeks 5 and 6 of RT (26). Hyperfractionation is associated with more severe acute oral toxicities, primarily mucositis. One animal experiment showed that at day 10 after RT, the oral size % of mice was 2, 5, 27, and 31 percent for 15, 18, 20, and 25 Gy RT (27). RIOM membrane inflammation and ulcer play a very important role in tissue injury caused by IL-1, TNF-α, and other inflammatory cytokines released from epithelial cells, blood vessels, and connective tissue; these can increase the wall permeability of capillaries and the numbers of inflammatory cells, such as myeloperoxidase-positive leukocytes and macrophages and neutrophil infiltration (28). Production of ROS is a key link in the aggravation of inflammatory injury. Sonis et al. (29, 30) showed that signal amplification is the core link in the development of RIOM into ulcer. Amplification of ROS and inflammatory cytokines occurs mainly through the following three steps: (1) activating the NF-κB pro-inflammatory pathway, stimulating target gene expression, and producing a large number of inflammatory cytokines, such as TNF-α, IL-1, and IL-6, which activate the ceramide pathway, thereby producing large amounts of sphingomyelinase and ceramide synthase and eventually causing more tissue damage and cell apoptosis; (2) fibronectin breakdown, which stimulates macrophages leading to activation of MMPs; (3) impaired mitochondria produce more ROS, which activates the NOD-like receptor pyrin domain-containing protein 3 (NLRP3) inflammasome pathway. NLRP3 activates caspase-1, which produces IL-1β and leads to apoptosis (31). In addition, the loss of a protective barrier in the basement membrane at the ulcer site increases the likelihood that gram-negative and yeast bacteria will develop secondary infections, which perpetuates inflammation and complicates existing inflammation (**Figure 1**).

Good oral hygiene can significantly reduce the risk of RIOM and is one of the most effective prevention methods, with only a few alternative effective treatments. Currently, symptomatic treatment is mostly adopted in clinical practice mainly including nutritional intervention, pain control, prevention and/or treatment of secondary infection (32–35). Many studies have proved that nutritional intervention improves acute radiation-induced oral mucosal response of HNC patients. Nutritional intervention can effectively maintain the nutritional status of patients with HNC treated with RT and promotes recovery from acute RIOM. It has also been pointed out that the protein level in the body during RT also affects the severity of oral mucositis, and low protein levels have negative effects on RIOM healing (17). Therefore, a growing number of physicians focus on the effects of early nutritional intervention on RIOM and on the nutritional status of patients with HNC.

### Malnutrition

The nutritional status of many HNC patients is at high risk of cachexia. RT or chemoradiotherapy also increases the risk of malnutrition. There is evidence that because of the location and treatment of the primary tumor, > 90% of patients have dysphagia, dry mouth, taste change, and oral mucositis, thereby affecting mouth opening and eating and resulting in symptoms of malnutrition (24, 36–38). Both acute and chronic malnutrition can lead to a decline in patients’ quality of life (39). Malnutrition and cachexia, which affect the course of RT treatment of patients and increase the risk of early death of patients, are considered as poor prognostic factors for HNC patients undergoing RT. Undoubtedly, the side effects caused by RT to the head and neck are key factors for malnutrition in patients. However, many other factors are important for the occurrence of malnutrition, such as smoking and drinking, and even psychological stress (40). A recent study suggested that the polymorphism SELP-2028 C/T of the P-selectin adhesion molecule gene in HNC patients undergoing RT could be used as a risk factor marker for malnutrition. P-selectin adhesion molecule plays an important role in activating leukocyte recruitment, promoting tumor invasion, and cancer cachexia at the site of inflammatory injury. CC homozygotes are four times more likely to be diagnosed with severe malnutrition and have a higher risk of early death than other genotype carriers (41). These results may provide the basis for early intervention in HNC patients undergoing RT.

Malnutrition has a huge effect on RT in patients with HNC. Therefore, it is necessary to assess the nutritional status during RT. Currently, the commonly used nutrition assessment tools include nutritional risk screening 2002 (NRS2002), malnutrition universal screening tool (MUST), mini nutrition assessment (MNA), and patient-generated subjective globe assessment (PG-SGA) (**Table 1**).

**Table 1:** The commonly used nutrition assessment tools.

	Composition	Assessment criteria
NRS2002	Disease score Nutritional status Age score	0~2: low nutritional risk >3:high nutritional risk, need nutrition intervention
MUST	BMI Weight loss Eating state	0: low nutritional risk 1: moderate nutritional risk 2: high nutritional risk, need nutrition intervention
MNA	Anthropometric assessment Global evaluation Dietetic evaluation Subjective assessment	≥24: normal 17–24: nutritional risk <17: malnutrition
PG-SGA	Weight loss Disease score Metabolic stress Physical examination Global assessment grading	A: normal B: nutritional risk C: severe malnutrition

NRS2002, nutritional risk screening 2002; MUST, malnutrition universal screening tool; MNA, mini nutrition assessment; PG-SGA, patient-generated subjective globe assessment; BMI, body mass index.

NRS2002 is a screening tool recommended by the European Society for Parenteral Nutrition (ESPEN) in 2002. It includes a disease severity score, an impaired nutritional status score, and an age score. If the three scores add up to > 3, the patient is at nutritional risk. With high reliability, NRS2002 can truly and objectively reflect the nutritional risk of tumor patients and is suitable for clinical use. It is a primary screening tool that has passed the evidence analysis standards of the American Association of Dietitians. The United States recommend NRS2002 for hospitalized patients with nutritional risk screening (42). NRS2002 is good for screening the nutritional status of elderly inpatients or cancer inpatients, especially for tumors affecting the digestive tract such as the oropharynx and esophagus (43). It has been reported that NRS2002 score is a simple and useful indicator for predicting long-term prognosis in patients with esophageal cancer after chemoradiotherapy (44). Peng et al. (45) screened 3,232 qualified patients to determine the application value of NRS2002 in patients with nasopharyngeal cancer. By analyzing the survival rate and quality of life of patients, they adjusted the NRS2002 screening tool and obtained a simpler and more clinically practical nutritional risk screening tool for nasopharyngeal cancer.

MUST was developed by the multidisciplinary malnutrition advisory group of the British association for parenteral nutrition and consisted of three assessments of body mass index (BMI), degree of body mass loss, and reduced dietary intake. A score > 2 indicates high nutritional risk. MUST is a simple and effective tool for nutritional risk assessment of cancer patients and has been widely accepted and effectively used by health professionals (46). In hospitalized cancer patients, MUST has the highest coincidence rate with NRS2002 and is a good identifier of patients at risk of prolonged hospitalization (47).

Developed according to subjective globe assessment, PG-SGA is a nutritional status assessment method specially designed for tumor patients and recommended by the American Dietitian Association. PG-SGA includes both patient self-assessment and medical personnel assessment. It is divided into three grades according to the obtained score: A, B, and C. Grades B or C indicate moderate or severe malnutrition, respectively. The incidence of malnutrition was high in patients with oral cancer before RT. Nutritional intervention for nasopharyngeal carcinoma patients undergoing concurrent chemoradiotherapy according to PG-SGA scores showed that the intervention significantly reduced acute radiation toxicity and improved patient nutritional status, quality of life, and treatment compliance (48). The use of PG-SGA to assess nutritional status before RT can be used as a prediction factor for RT response. PG-SGA nutritional score < 9 was associated with better local control and acute toxicity in patients undergoing radical head and neck cancer treatment (49). PG-SGA can also assess the nutritional status of patients with enteral nutrition and head and neck cancer treated with gastrostomy, especially in patients with impaired language skills (50).

MNA is a new evaluation method of human nutritional status established and developed by Vellas et al. (51–54) in the 1990s. It includes anthropometric measurements, overall evaluation, a dietary questionnaire, and subjective evaluation. The method is simple and shows good linear correlation with the human body composition evaluation method.

PG-SGA is more focused on the evaluation of chronic nutritional changes than NRS2002. MUST is specifically used to assess the risk of protein-calorie malnutrition with high accuracy and reliability. PG-SGA was significantly consistent with MNA, however, consistency between PG-SGA and NRS-2002 was moderate (55).

## Clinical Intervention for RIOM and Malnutrition in HNC Patients

### Medication

There is still no specific treatment for RIOM. To promote healing from RIOM, the treatment has been focused on relieving the pain and inhibiting oxidative stress and the inflammatory response in patients (**Table 2**). Anesthetics and analgesics are often administered to relieve the pain caused by RIOM. According to the evidence-based clinical practice guidelines on mucositis published by the mucositis research group of the Multinational Association of support Care in Cancer/International Society of Oral Oncology (MASCC/ISOO), morphine mouthwash and doxepin mouthwash are recommended for HNC patients undergoing RT (33, 56, 57). Studies have also shown that introduction of low-dose controlled-release oxycodone in the early stage of moderate pain in patients with RT and chemotherapy for nasopharyngeal cancer can help to reduce total dose, provide better pain control, reduce weight loss, and improve quality of life (58–60). Anti-inflammatory therapy is also a common clinical treatment. The MASCC/ISOO mucositis guidelines have recommended the use of benzydamine mouthwash to prevent oral mucositis in HNC patients (61). A number of recent studies have also reported that some other drugs have been effective in reducing oxidative stress and inflammatory responses to RIOM. Rosiglitazone, a peroxisome proliferator activated-receptor (PPAR) gamma agonist stimulation drug, has anti-inflammatory and anti-fibrosis effects. It inhibits the growth of irradiation-induced transforming growth factor beta (TGF-β) and NF-κB p65 subunit proteins and enhances the expression of catalase to protect the oral mucosa without affecting the efficacy of RT (62). Thalidomide, an NF-κB inhibitor, significantly improved mucosal tissue in irradiated mice, although the underlying mechanism still requires further study (63). Other drugs, such as amiforstine and gliclazide, reduce oxidative stress and inflammation (64, 65). Growth factors and cytokines are also used to treat radiation mucositis. RT can induce apoptosis of proliferating basal cells, and growth factors such as epidermal growth factor and keratinocyte growth factor (KGF) accelerate the conversion rate of epithelial cells and contribute to the regeneration of oral mucosal cells (66, 67). In addition, some drugs have been reported to promote healing of RIOM. Luo et al. (68) fused Smad7, multifunctional protein, with a cell-penetrating Tat tag (Tat-smad7), which was applied to the oral mucosa of RT mice at the onset of oral mucositis. The results suggested that short-term application of Tat-Smad7 promoted oral mucositis healing without affecting the cytotoxic effect of RT on cancer cells. Both multivitamin B and GeneTime (R) have been used in the treatment of oral inflammation. There is evidence that the combination of multivitamin B and GeneTime (R) is more effective for the treatment of RIOM and can reduce the healing time of ulcer (69).

**Table 2:** Drugs commonly used to treat RIOM.

Drugs category	Drugs	Mechanism	Treatment effect	Reference
Analgesic drugs	Morphine and Doxepin mouthwash	Anesthesia and analgesia	Reduce the pain caused RIOM Increase eating	(33, 56, 57)
	Oxycodone			(58–60)
Anti-inflammatory drugs	Benzydamine mouthwash	Local anti-inflammatory	Prevent RIOM	(61)
	Rosiglitazone	PPAR-γ agonists, Inhibition of TGF-β and NF-κB p65 expression	Protect normal oral mucosa and antitumor	(62)
	Thalidomide	Inhibition of NF-κB	Reduce inflammatory response	(63)
	amiforstine and gliclazide	Reduce oxidative stress and inflammation caused by 5-fluorouracil	Accelerate the recovery oral mucositis caused by 5-fluorouracil	(64, 65)
Cytogenetic drugs	epidermal growth factor and keratinocyte growth factor	promote the regeneration of oral mucosal cells	promote the healing of RIOM	(66, 67)
Others	Tat-smad7	Reduce TGF-β and NF-κB signaling pathways	Reduce inflammatory response and promote the healing of RIOM	(68)
	multivitamin B + Gene Time(R)	Promote the synthesis of DNA, RNA and hydroxyproline	Shorten the healing time of ulcer	(69)

PPAR, peroxisome proliferator activated-receptor; TGF, transforming growth factor; NF-κB, nuclear factor-kappa B; RIOM, radiation-induced oral mucositis.

### Oral Health and Photobiomodulation Therapy (PBMT)

Although RIOM is not caused by pathogens, destruction of the mucosal barrier facilitates invasion of pathogens. Infection complicates the damage of mucositis. The abundance of a variety of gram-negative bacteria (*Fusobacterium, Haemophilus, Tannerella, Porphyromonas, and Eikenella*) in the oral mucosa may influence susceptibility of patients to RIOM (70). Therefore, oral health intervention is necessary. As a common superficial oral infection in cancer patients, *Candida* colonization in the oral mucosa may delay RIOM healing. Miconazole, an antifungal drug, is expected to reduce the length of hospital stay for RIOM and the use of morphine in patients (71). In addition, probiotics such as *Bacteroidetes* and *Bifidobacteria* can significantly increase the number and activity of immune cells and are beneficial for RIOM. Studies have shown that a combination of probiotics can significantly enhance the immune response of patients and reduces the severity of RIOM by changing the intestinal microbiota (72–74).

PBMT, which is recommended by MASCC/ISOO for tumor support therapy, can also be used to prevent and treat RIOM (75). PBMT can improve the quality of life, effectively control RIOM, and reduce the incidence and associated costs of RIOM (76, 77). Currently, the best studied PBMT includes low-level laser therapy (LLLT) and photodynamic therapy (PT). One study evaluated the efficacy of LLLT in the prevention and treatment of oral and oropharyngeal mucositis in patients with oral squamous cell carcinoma because of chemoradiotherapy. After 5 weeks of treatment, 72.7% of the mucosa in the laser group was normal (Grade 0), 20.0% of the control group was Grade 0, and 40.0% of the control group was Grade 2. The effect of LLLT in reducing the incidence and severity of mucositis is significant (78). Both LLLT and PT stimulate the expression of basic fibroblast growth factor, TGF-β, and platelet-derived growth factor. The increase in basic fibroblast growth factor and platelet-derived growth factor levels because of PT is more obvious than that because of LLLT, and the effect of PT appears to be more significant than that of LLLT (79).

### Nutrition Intervention

HNC, especially head and neck squamous cell carcinoma, is usually found in advanced stages. The nutritional status of patients at the time of admission are affected to varying degrees by chemoradiotherapy, which can also cause or aggravate malnutrition in patients. RIOM over Grade 3 can aggravate the degree of malnutrition in patients with locally advanced nasopharyngeal carcinoma during RT (80). Many studies have shown that nutritional intervention can not only reduces the risk and severity of RIOM and improve the nutritional status of patients with HNC, but also improve patients’ tolerance to RT and quality of life and enhance treatment efficacy (81–83). The best application for nutritional intervention is through oral intake. Oral nutrition is the first choice in patients who can eat. However, because of complications such as oral mucositis caused by the tumor itself or chemoradiotherapy, a patient’s swallowing function is greatly affected. Currently, the main nutritional interventions are enteral nutrition and parenteral venous nutrition. There are many complications of intravenous nutrition, and enteral nutrition including nasogastric tube feeding and gastrostomy feeding is highly recommended in the clinic. Corry et al. (84) studied the effects of gastrostomy and nasogastric tube feeding in HNC patients treated with RT or chemoradiotherapy. The authors found that patients who underwent percutaneous endoscopic gastrostomy gained significantly more weight than those fed through a nasogastric tube. However, after 6 months of treatment, there was no difference in weight gain between the two groups, and the associated costs were 10 times higher in the gastrostomy group than in the nasogastric tube group. The duration of enteral nutrition in the gastrostomy group was significantly longer than that in the nasogastric tube group although there was no significant difference in pulmonary infection rate between the two groups. Current nutritional interventions are mostly initiated when patients’ food intake is affected, although the nutritional status cannot be significantly reversed during RT (80). Therefore, early nutritional intervention is attracting more attention. A study of the effectiveness of preventive percutaneous endoscopic gastrostomy (PEG) demonstrated that only 22% of patients lost > 10% of their initial body weight. The most common complication was a minor perioral infection associated with the use of proton pump inhibitors before PEG placement.

### Early Nutritional Intervention

There are different views on the timing of nutritional intervention in clinical practice. Nutritional intervention usually begins when patients with RIOM or severe gastrointestinal reactions have restricted feeding. However, the nutritional status of patients is difficult to improve significantly. Some prospective studies have demonstrated the significant effects of early nutritional intervention on RIOM and nutritional status in HNC patients undergoing RT (85, 86). Early nutritional interventions can lead to higher serum albumin and hemoglobin levels in HNC patients (87). Plasma albumin, which reflects the level of human protein, is an important nutrient for human body. The amino acid produced by the breakdown of plasma albumin can be used for synthesis of tissue proteins, energy supply, or conversion to other nitrogenous substances. And the decrease in hemoglobin reflects deficiencies in many nutrients, such as iron and vitamins (especially B12). Early nutritional intervention by a multidisciplinary nutritional support team improved body weight loss rate, mucositis, albumin level, and hospital length of stay, which might lead to better clinical outcomes (88). Therefore, early nutritional intervention may contribute to improve malnutrition of patients. Meng et al. (89) divided 78 cases of nasopharyngeal cancer patients into early nutritional intervention group and late nutritional intervention group respectively. Early nutritional intervention began at the beginning of chemoradiotherapy, while late nutritional intervention began when the side effects of RT were evident. Both groups had reduced weight at the end of chemoradiotherapy and 3 months later. However, 3 months after finishing chemoradiotherapy, the early group began to regain weight, while the late group continued to lose weight. The weight, BMI, albumin, and prealbumin levels in the early group were lower than those in the late group during and after radiation therapy. Early nutritional intervention can reduce the incidence and level of severe oral mucositis (90).

Systematic organization of early nutritional therapy in HNC patients is absolutely essential. Early nutritional intervention can not only prevent and treat RIOM, but also effectively improve the nutritional status of patients and improve their tolerance to chemoradiotherapy and overall survival rates (91). According to the PG-SGA score, the number of patients with good nutrition was higher in the early stage of treatment than in the late stage. In addition, many patients had to stop the course of RT because of RIOM and malnutrition. The benefits of early nutritional intervention for RIOM and malnutrition are also reflected in the lower incidence of RT interruption, which ensures smooth treatment (85). There was a linear correlation between the percentage of weight loss in HNC patients and the days delayed in RT process from before the end of radiotherapy (92). Early nutritional interventions, including oral feeding, nasogastric tube, and gastrostomy, can significantly improve weight loss and the interruption/delay of radiotherapy process and reduce the probability of accidental hospitalization (87, 93–97). In patients with enteral nutrition contraindications, even early 7-day supplemental parenteral nutrition improved their body composition at nutritional risk in the absence of any relevant clinical complications (98). A prospective study by Wei et al. (86) divided 54 HNC patients into an early nutritional group and an advanced nutritional intervention group. The results showed that the incidence of oral mucositis was significantly lower in the early group than in the late group. The nutritional status of these patients was assessed at week 4 and week 7, and the weight and BMI declines were more pronounced in the late group than in the early group. Plasma albumin, hemoglobin, and prealbumin levels, and total lymphocyte counts were significantly lower in both groups after week 7 of RT.

These prospective studies show that early nutritional intervention improves oral mucositis and nutritional status of patients with head and neck malignant tumors who undergo chemoradiotherapy (**Table 3**). In addition, it prevents malnutrition-related complications in tumor patients, avoids the interruption of RT, and improves the long-term quality of life of patients, which has broad implications. Further research is required to support the use of early nutritional support in patients undergoing RT for HNC.

**Table 3:** Influence of early nutritional intervention on patients with head and neck radiotherapy.

Author	Year	Patients		Treatment	Result	p
		ENG	CG			
Meng et al. (89)	2019	46	32	CRT	CT/CRT break:10.9% VS. 25.0%	0.017
					days of CRT delayed for toxicity: 2.2 ± 1.8 VS. 3.1 ± 3.2	0.033
					rate of patients with unplanned hospitalizations:13.0% VS. 31.3%	0.009
					advanced oral mucositis (3, 4):13.0% VS. 21.9%	0.028
Paccagnella et al. (92)	2010	33	33	CRT	weight loss: −2.4 ± 8.2% VS. −9.6 ± 8.1%	0.0077
					CT/CRT break:30.3% VS. 63.6%	0.007
					days of CRT delayed for toxicity: 4.4 ± 5.2 VS. 7.6 ± 6.5	0.038
					rate of patients with unplanned hospitalizations: 16.1% VS. 41.4%	0.030
Piquet et al. (96)	2002	45	45	RT	weight loss: 3.5 ± 0.7% VS. 6.1 ± 0.7%	<0.01
					hospital admission for dehydration: 0% VS. 18.0%	<0.01
Wang et al. (87)	2012	35	23	CCRT	weight loss:−1.68 ± 6.33% VS. −6.55 ± 7.28%	<0.01
					CT/CRT break: 28.6% VS. 73.9%	0.010
					albumin level change: −15.05 ± 10.41% VS. −27.38 ± 11.41%	0.002
Wei et al. (86)	2020	28	26	CT/CRT	weight loss: −5.64 ± 2.54kg VS. −8.77 ± 1.61kg	<0.001
					albumin level change: −4.79 ± 3.69g/L VS. −7.09 ± 3.39g/L	<0.001
					hemoglobin loss: −12.96 ± 19.83 g/L VS. -14.81 ± 24.47 g/L	<0.001
					advanced oral mucositis (3, 4):17.9% VS. 50.0%	0.012
Isenring et al. (99)	2004	29	31	RT	weight loss: −0.4kg VS. −4.7kg	<0.001
					NI are beneficial to global QoL	0.009
González-Rodríguez et al. (97)	2020	135	39	RT	malnutrition: 31.9% VS. 69.5%	0.0001
					emergency visits 0. 75 VS. 1.1 episodes per patient	0.021
					hospitalizations: 29% VS. 59%	0.044
Kono et al. (88)	2020	32	61	CCRT	Grade III mucositis: 25.0% VS. 70.0%	0.006

ENG, early nutritional intervention group; CG, control group; RT, radiotherapy; CRT, chemoradiotherapy; CCRT, concurrent chemoradiotherapy; NI, nutrition intervention; QoL, quality of life. Values of p < 0.05 were considered significant.

## Conclusion

In conclusion, radiation-induced oral mucositis and malnutrition have a significant effect on the course and recovery in HNC patients treated with RT. Many studies have shown that early nutritional intervention improves oral mucositis and nutritional status of patients. Although a number of prospective randomized trials have been conducted worldwide to assess the effects of early nutritional interventions on quality of life in cancer patients, comparison and evaluation of the effects of different nutritional intervention timings on the nutritional status of HNC patients undergoing RT have not been reported. More in-depth research is still required.

## Author Contributions

Study concepts: ZZ. Study design: XZ. Data acquisition: QZ. Quality control of data and algorithms: YZ. Data analysis and interpretation: SL. Statistical analysis: ZL. Manuscript preparation: ZZ and LM. Manuscript editing: YX and XJ. Manuscript review: YX and XJ. All authors contributed to the article and approved the submitted version.

## Funding

This work was supported by the National Natural Science Foundation of China (Grant number 81570344); National Key R&D Program of China (Grant number 2017YFC0112100); the Education Department Foundation of Jilin Province (Grant number JJKH20201036KJ); the Health and Family Planning Commission of Jilin Province Foundations (Grant number 2016Q034 and 2017J11) and the Jilin Provincial Science and Technology Foundations (Grant number 20180414039GH and 20190201200JC).

## Conflict of Interest

The authors declare that the research was conducted in the absence of any commercial or financial relationships that could be construed as a potential conflict of interest.

## Abbreviations

ADA, American Dietitian Association; BMI, Body Mass Index; ESPEN, European Society for Parenteral Nutrition; IL, interleukin; KGF, keratinocyte growth factor; LLLT, low-level laser therapy; MASCC/ISOO, the Multinational Association of Support Care in Cancer/International Society of Oral Oncology; MMPs, matrix metalloproteinases; MNA, mini nutrition assessment; MUST, malnutrition universal screening tool; NF-κB, nuclear factor-kappaB; NRS2002, nutritional risk screening 2002; PBMT, photobiomodulation therapy; PEG, percutaneous endoscopic gastrostomy; PG-SGA, patient-generated subjective globe assessment; PPAR, peroxisome proliferator-activated receptor; PT, photodynamic therapy; RIOM, radiation-induced oral mucositis; ROS, reactive oxygen species; RT, radiotherapy; RTOG, Radiation Therapy Oncology Group; TGF-β, transforming growth factor; TNF-α, tumor necrosis factor alpha.

## References

[1] Marur S Forastiere AA Head and Neck Squamous Cell Carcinoma: Update on Epidemiology, Diagnosis, and Treatment. Mayo Clin Proc (2016) 91:386–96. 10.1016/j.mayocp.2015.12.017 26944243

[2] Torre LA Bray F Siegel RL Ferlay J Lortet-Tieulent J Jemal A Global Cancer Statistics, 2012. Ca-a Cancer J For Clin (2015) 65:87–108. 10.3322/caac.21262 25651787

[3] Capuano G Gentile PC Bianciardi F Tosti M Palladino A Di Palma M Prevalence and influence of malnutrition on quality of life and performance status in patients with locally advanced head and neck cancer before treatment. Supportive Care In Cancer (2010) 18:433–7. 10.1007/s00520-009-0681-8 19562384

[4] Capuano G Grosso A Gentile PC Battista M Bianciardi F Di Palma A Influence of weight loss on outcomes in patients with head and neck cancer undergoing concomitant chemoradiotherapy. Head Neck (2008) 30:503–8. 10.1002/hed.20737 18098310

[5] De Luis DA Izaola O Aller R Nutritional status in head and neck cancer patients. Eur Rev For Med And Pharmacol Sci (2007) 11:239–43. 17876958

[6] Beaver ME Matheny KE Roberts DB Myers JN Predictors of weight loss during radiation therapy. Otolaryngol Head And Neck Surg (2001) 125:645–8. 10.1067/mhn.2001.120428 11743469

[7] Elting LS Cooksley CD Chambers MS Garden AS Risk, outcomes, and costs of radiation-induced oral mucositis among patients with head-and-neck malignancies. Int J Radiat Oncol Biol Phys (2007) 68:1110–20. 10.1016/j.ijrobp.2007.01.053 17398022

[8] Muanza TM Cotrim AP McAuliffe M Sowers AL Baum BJ Cook JA Evaluation of radiation-induced oral mucositis by optical coherence tomography. Clin Cancer Res (2005) 11:5121–7. 10.1158/1078-0432.CCR-05-0403 16033826

[9] Jakob M Manz M Schroeck A Bootz F Eichhorn K Analysis of Quality of Life Outcome for Nasopharyngeal Carcinoma Patients After Treatment. Laryngo-Rhino-Otologie (2013) 92:244–50. 10.1055/s-0032-1330020 23296462

[10] Campos MI Campos CN Aarestrup FM Aarestrup BJ Oral mucositis in cancer treatment: Natural history, prevention and treatment. Mol Clin Oncol (2014) 2:337–40. 10.3892/mco.2014.253 PMC3999143 24772297

[11] Rose-Ped AM Bellm LA Epstein JB Trotti A Gwede C Fuchs HJ Complications of radiation therapy for head and neck cancers - The patient’s perspective. Cancer Nurs (2002) 25:461–7. 10.1097/00002820-200212000-00010 12464838

[12] Kubrak C Martin L Gramlich L Scrimger R Jha N Debenham B Prevalence and prognostic significance of malnutrition in patients with cancers of the head and neck. Clin Nutr (2020) 39:901–9. 10.1016/j.clnu.2019.03.030 31000341

[13] Deng J He Y Sun X-S Li J-M Xin M-Z Li W-Q Construction of a comprehensive nutritional index and its correlation with quality of life and survival in patients with nasopharyngeal carcinoma undergoing IMRT: A prospective study. Oral Oncol (2019) 98:62–8. 10.1016/j.oraloncology.2019.09.014 31541928

[14] Ma L Wu T Pan J Kong X Guo Q Yang L The correlation between the comprehensive nutrition index and quality of life of patients with nasopharyngeal carcinoma treated by intensity-modulated radiotherapy. Nutr Cancer (2014) 66:152–8. 10.1080/01635581.2014.853815 24328938

[15] Talwar B Donnelly R Skelly R Donaldson M Nutritional management in head and neck cancer: United Kingdom National Multidisciplinary Guidelines. J Laryngol And Otol (2016) 130:S32–40. 10.1017/S0022215116000402 PMC4873913 27841109

[16] Gillison ML Current topics in the epidemiology of oral cavity and oropharyngeal cancers. Head Neck (2007) 29:779–92. 10.1002/hed.20573 17230556

[17] Zahn KL Wong G Bedrick EJ Poston DG Schroeder TM Bauman JE Relationship of protein and calorie intake to the severity of oral mucositis in patients with head and neck cancer receiving radiation therapy. Head Neck (2012) 34:655–62. 10.1002/hed.21795 21692134

[18] Kelvianto A Witjaksono F Sekarutami SM Protein Intake, Prognostic Nutritional Index and Quality of Life in Head and Neck Cancer Patients Undergoing Radiotherapy. Indonesian Biomed J (2019) 11:70–7. 10.18585/inabj.v11i1.570

[19] Cereda E Turri A Klersy C Cappello S Ferrari A Filippi AR Whey protein isolate supplementation improves body composition, muscle strength, and treatment tolerance in malnourished advanced cancer patients undergoing chemotherapy. Cancer Med (2019) 8:6923–32. 10.1002/cam4.2517 PMC6853834 31568698

[20] Kostler WJ Hejna M Wenzel C Zielinski CC Oral mucositis complicating chemotherapy and/or radiotherapy: Options for prevention and treatment. Ca-a Cancer J For Clin (2001) 51:290–315. 10.3322/canjclin.51.5.290 11577493

[21] Cox JD Stetz J Pajak TF Toxicity criteria of the Radiation Therapy Oncology Group (RTOG) and the European Organization for Research and Treatment of Cancer (EORTC). Int J Radiat Oncol biol Phys (1995) 31:1341–6. 10.1016/0360-3016(95)00060-C 7713792

[22] Feller L Essop R Wood NH Khammissa RAG Chikte UME Meyerov R Chemotherapy- and radiotherapy-induced oral mucositis: pathobiology, epidemiology and management. SADJ J South Afr Dental Assoc = tydskrif van die Suid-Afrikaanse Tandheelkundige Vereniging (2010) 65:372–4. 21133051

[23] Sonis ST Mucositis as a biological process: a new hypothesis for the development of chemotherapy-induced stomatotoxicity. Oral Oncol (1998) 34:39–43. 10.1016/S1368-8375(97)00053-5 9659518

[24] Russo G Haddad R Posner M Machtay M Radiation treatment breaks and ulcerative mucositis in head and neck cancer. Oncologist (2008) 13:886–98. 10.1634/theoncologist.2008-0024 18701763

[25] Luo D-H Hong M-H Guo L Cao K-J Deng M-Q Mo H-Y Analysis of oral mucositis risk factors during radiotherapy for nasopharyngeal carcinoma patients and establishment of a discriminant model. Ai zheng = Aizheng = Chin J Cancer (2005) 24:850–4. 16004814

[26] Chen SC Lai YH Huang BS Lin CY Fan KH Chang JT Changes and predictors of radiation-induced oral mucositis in patients with oral cavity cancer during active treatment. Eur J Oncol Nurs (2015) 19:214–9. 10.1016/j.ejon.2014.12.001 25586214

[27] Maria OM Syme A Eliopoulos N Muanza T Single-Dose Radiation-Induced Oral Mucositis Mouse Model. Front Oncol (2016) 6:154. 10.3389/fonc.2016.00154 27446800 PMC4921469

[28] Chamberlain G Fox J Ashton B Middleton J Concise review: Mesenchymal stem cells: Their phenotype, differentiation capacity, immunological features, and potential for homing. Stem Cells (2007) 25:2739–49. 10.1634/stemcells.2007-0197 17656645

[29] Sardaro N Della Vella F Incalza MA Di Stasio D Lucchese A Contaldo M Oxidative Stress and Oral Mucosal Diseases: An Overview. In Vivo (2019) 33:289–96. 10.21873/invivo.11474 PMC6506298 30804105

[30] Sonis ST Elting LS Keefe D Peterson DE Schubert M Hauer-Jensen M Perspectives on cancer therapy-induced mucosal injury - Pathogenesis, measurement, epidemiology, and consequences for patients. Cancer (2004) 100:1995–2025. 10.1002/cncr.20162 15108222

[31] Redding SW Cancer therapy-related oral mucositis. J Dental Educ (2005) 69:919–29. 10.1002/j.0022-0337.2005.69.8.tb03989.x 16081575

[32] Elad S The MASCC/ISOO Mucositis Guidelines 2019 Update: introduction to the first set of articles. Supportive Care Cancer (2019) 27:3929–31. 10.1007/s00520-019-04895-x 31286226

[33] Lalla RV Bowen J Barasch A Elting L Epstein J Keefe DM MASCC/ISOO Clinical Practice Guidelines for the Management of Mucositis Secondary to Cancer Therapy. Cancer (2014) 120:1453–61. 10.1002/cncr.28592 PMC4164022 24615748

[34] Lalla RV Ashbury FD The MASCC/ISOO Mucositis Guidelines: dissemination and clinical impact. Supportive Care Cancer (2013) 21:3161–3. 10.1007/s00520-013-1924-2 23942597

[35] Lalla RV The MASCC/ISOO Mucositis Guidelines Update: introduction to the first set of articles. Supportive Care Cancer (2013) 21:301–2. 10.1007/s00520-012-1660-z 23161340

[36] Gellrich NC Handschel J Holtmann H Kruskemper G Oral cancer malnutrition impacts weight and quality of life. Nutrients (2015) 7:2145–60. 10.3390/nu7042145 PMC4425137 25825828

[37] Rosenthal DI Mendoza TR Chambers MS Burkett VS Garden AS Hessell AC Anderson symptom inventory-head and neck module, a patient-reported outcome instrument, accurately predicts the severity of radiation-induced mucositis. Int J Radiat Oncol Biol Phys (2008) 72:1355–61. 10.1016/j.ijrobp.2008.02.072 18501527

[38] Hofman M Ryan JL Figueroa-Moseley CD Jean-Pierre P Morrow GR Cancer-related fatigue: the scale of the problem. Oncologist (2007) 12 Suppl 1:4–10. 10.1634/theoncologist.12-S1-4 17573451

[39] Crowder SL Douglas KG Pepino MY Sarma KP Arthur AE Nutrition impact symptoms and associated outcomes in post-chemoradiotherapy head and neck cancer survivors: a systematic review. J Of Cancer Survivorship (2018) 12:479–94. 10.1007/s11764-018-0687-7 29556926

[40] Zhu C Wang B Gao Y Ma X Prevalence and relationship of malnutrition and distress in patients with Cancer using questionnaires. BMC Cancer (2018) 18(1):1272. 10.1186/s12885-018-5176-x 30567507 PMC6299972

[41] Powrozek T Mlak R Brzozowska A Mazurek M Golebiowski P Malecka-Massalska T Relationship Between-2028 C/T SELP Gene Polymorphism, Concentration of Plasma P-Selectin and Risk of Malnutrition in Head and Neck Cancer Patients. Pathol Oncol Res (2019) 25:741–9. 10.1007/s12253-018-00578-w 30617759

[42] Kondrup J Allison SP Elia M Vellas B Plauth M ESPEN guidelines for nutrition screening 2002. Clin Nutr (2003) 22:415–21. 10.1016/S0261-5614(03)00098-0 12880610

[43] Liu W Gao L Huang X Luo J Zhang S Wang K Pretreatment nutritional risk as a prognostic factor in head and neck cancer patients receiving radiotherapy or chemoradiotherapy. Asia Pacific J Clin Nutr (2019) 28:223–9. 10.6133 / 10.6133/apjcn.201906_28(2).0003 31192550

[44] Wang J Yu B Ye Y Shen J Ding N Tang H Predictive Value of Nutritional Risk Screening 2002 and Prognostic Nutritional Index for Esophageal Cancer Patients Undergoing Definitive Radiochemotherapy. Nutr And Cancer-An Int J (2018) 70:879–85. 10.1080/01635581.2018.1470656 30273001

[45] Peng H Chen B-B Tang L-L Chen L Li W-F Zhang Y Prognostic value of nutritional risk screening 2002 scale in nasopharyngeal carcinoma: A large-scale cohort study. Cancer Sci (2018) 109:1909–19. 10.1111/cas.13603 PMC5989749 29624824

[46] Coronha AL Lourenco C Ferreira M Reis N Almeida R Boleo-Tome C RESEARCH TRAINING IN NUTRITION Relevance for medical clinical pratice. Acta Med Portuguesa (2011) 24:885–92. 22713181

[47] Amaral TF Antunes A Cabral S Alves P Kent-Smith L An evaluation of three nutritional screening tools in a Portuguese oncology centre. J Hum Nutr Dietetics (2008) 21:575–83. 10.1111/j.1365-277X.2008.00917.x 19017102

[48] Peng S Huang Y Li H Yang R Zeng Q Application of Nutritional Intervention Assessed By the PGSGA in Nasopharyngeal Carcinoma Patients Undergoing Chemoradiotherapy. Int J Radiat Oncol Biol Phys (2019) 105:E559–60. 10.1016/j.ijrobp.2019.06.1308

[49] Bahl A Elangovan A Kaur S Verman R Oinam AS Ghoshal S Pre-Treatment Nutritional Status and Radiotherapy Outcome in Patients with Locally Advanced Head and Neck Cancers. Gulf J Oncol (2017) 1:61–3. 29019332

[50] Correira Pereira MA Santos CA Almeida Brito J Fonseca J Scored Patient-Generated Subjective Global Assessment, albumin and transferrin for nutritional assessment of gastrostomy fed head or neck cancer patients. Nutricion hospitalaria (2014) 29:420–6. 10.3305/nh.2014.29.2.7066 24528363

[51] Vellas B Villars H Abellani G Soto ME Rolland Y Guigoz Y Overview of the MNA (R) - Its history and challenges. J Nutr Health Aging (2006) 10:456–63. 17183418

[52] Vellas B Guigoz Y Baumgartner M Garry PJ Lauque S Albarede JL Relationships between nutritional markers and the mini-nutritional assessment in 155 older persons. J Am Geriatrics Soc (2000) 48:1300–9. 10.1111/j.1532-5415.2000.tb02605.x 11037019

[53] Vellas B Guigoz Y Garry PJ Nourhashemi F Bennahum D Lauque S The mini nutritional assessment (MNA) and its use in grading the nutritional state of elderly patients. Nutrition (1999) 15:116–22. 10.1016/S0899-9007(98)00171-3 9990575

[54] Guigoz Y Vellas B Garry PJ Assessing the nutritional status of the elderly: The Mini Nutritional Assessment as part of the geriatric evaluation. Nutr Rev (1996) 54:S59–65. 10.1111/j.1753-4887.1996.tb03793.x 8919685

[55] Demirel B Atasoy BM Comparison of Three Nutritional Screening Tools to Predict Malnutrition Risk and Detect Distinctions Between Tools in Cancer Patients Receiving Radiochemotherapy. Nutr Cancer-An Int J (2018) 70:867–73. 10.1080/01635581.2018.1491606 30273006

[56] Saunders DP Rouleau T Cheng K Yarom N Kandwal A Joy J Mascc, and Isoo, Systematic review of antimicrobials, mucosal coating agents, anesthetics, and analgesics for the management of oral mucositis in cancer patients and clinical practice guidelines. Supportive Care Cancer (2020) 28:2473–84. 10.1007/s00520-019-05181-6 32052137

[57] Rubenstein EB Peterson DE Schubert M Clinical practice guidelines for the prevention and treatment of cancer therapy-induced oral and gastrointestinal mucositis (vol 100(Supplement 9), pg 2026, 2004). Cancer (2004) 101:1921–1. 10.1002/cncr.20664 15108223

[58] Hua X Chen LM Zhu Q Hu W Lin C Long ZQ Efficacy of controlled-release oxycodone for reducing pain due to oral mucositis in nasopharyngeal carcinoma patients treated with concurrent chemoradiotherapy: a prospective clinical trial. Support Care Cancer (2019) 27:3759–67. 10.1007/s00520-019-4643-5 PMC6726700 30712098

[59] Guo S-P Wu S-G Zhou J Feng H-X Li F-Y Wu Y-J Transdermal fentanyl for pain due to chemoradiotherapy-induced oral mucositis in nasopharyngeal cancer patients: evaluating efficacy, safety, and improvement in quality of life. Drug Design Dev Ther (2014) 8:497–502. 10.2147/DDDT.S60187 PMC4026399 24872680

[60] Caraceni A Hanks GR Kaasa S Bennett MI Brunelli C Cherny N Epcrc, and Eapc, Use of opioid analgesics in the treatment of cancer pain: evidence-based recommendations from the EAPC. Lancet Oncol (2012) 13:E58–68. 10.1016/S1470-2045(12)70040-2 22300860

[61] Ranna V Cheng KKF Castillo DA Porcello L Vaddi A Lalla RV Development of the MASCC/ISOO clinical practice guidelines for mucositis: an overview of the methods. Supportive Care Cancer (2019) 27:3933–48. 10.1007/s00520-019-04891-1 31286227

[62] Mangoni M Sottili M Gerini C Desideri I Bastida C Pallotta S A PPAR gamma agonist protects against oral mucositis induced by irradiation in a murine model. Oral Oncol (2017) 64:52–8. 10.1016/j.oraloncology.2016.11.018 28024724

[63] Frings K Gruber S Kuess P Kleiter M Doerr W Modulation of radiation-induced oral mucositis by thalidomide. Strahlentherapie Und Onkologie (2016) 192:561–8. 10.1007/s00066-016-0989-5 27282278

[64] Mafra C Vasconcelos RC de Medeiros C Leitao RFD Brito GAD Costa DVD Gliclazide Prevents 5-FU-Induced Oral Mucositis by Reducing Oxidative Stress, Inflammation, and P-Selectin Adhesion Molecules. Front Physiol (2019) 10:327. 10.3389/fphys.2019.00327 30971955 PMC6445135

[65] Barbosa SCM Pereira VBM Wong DVT Santana APM Lucetti LT Carvalho LL Amifostine reduces inflammation and protects against 5-fluorouracil-induced oral mucositis and hyposalivation. Braz J Med Biol Res (2019) 52(3):e8251. 10.1590/1414-431x20188251 30810625 PMC6393848

[66] Riley P Glenny AM Worthington HV Littlewood A Fernandez Mauleffinch LM Clarkson JE Interventions for preventing oral mucositis in patients with cancer receiving treatment: cytokines and growth factors. Cochrane Database Syst Rev (2017) 11:Cd011990. 10.1002/14651858.CD011990.pub2 29181845 PMC6486203

[67] Sonis ST The pathobiology of mucositis. Nat Rev Cancer (2004) 4:277–84. 10.1038/nrc1318 15057287

[68] Luo J Bian L Blevins MA Wang D Liang C Du D Smad7 Promotes Healing of Radiotherapy-Induced Oral Mucositis without Compromising Oral Cancer Therapy in a Xenograft Mouse Model. Clin Cancer Res (2019) 25:808–18. 10.1158/1078-0432.CCR-18-1081 PMC6335168 30185419

[69] Sun H Zhu X Li D Cheng T Effects of a compound vitamin B mixture in combination with GeneTime (R) on radiation-induced oral mucositis. J Int Med Res (2019) 47:2126–34. 10.1177/0300060519831171 PMC6567777 30938568

[70] Vesty A Gear K Biswas K Mackenzie BW Taylor MW Douglas RG Oral microbial influences on oral mucositis during radiotherapy treatment of head and neck cancer. Supportive Care Cancer (2020) 28:2683–91. 10.1007/s00520-019-05084-6 31650293

[71] Orvain C Moles-Moreau MP Francois S Mercier M Moal F Hamel JF Miconazole mucoadhesive buccal tablet in high-dose therapy with autologous stem cell transplantation (HDT/ASCT)-induced mucositis. Supportive Care Cancer (2015) 23:359–64. 10.1007/s00520-014-2365-2 25084742

[72] Jiang C Wang H Xia C Dong Q Chen E Qiu Y A randomized, double-blind, placebo-controlled trial of probiotics to reduce the severity of oral mucositis induced by chemoradiotherapy for patients with nasopharyngeal carcinoma. Cancer (2019) 125:1081–90. 10.1002/cncr.31907 30521105

[73] Shu ZK Li PJ Yu BQ Huang S Chen YY The effectiveness of probiotics in prevention and treatment of cancer therapy-induced oral mucositis: A systematic review and meta-analysis. Oral Oncol (2020) 102:104559. 10.1016/j.oraloncology.2019.104559 31923856

[74] Al-Qadami G Van Sebille Y Le H Bowen J Gut microbiota: implications for radiotherapy response and radiotherapy-induced mucositis. Expert Rev Gastroenterol Hepatol (2019) 13:485–96. 10.1080/17474124.2019.1595586 30907164

[75] Zadik Y Arany PR Fregnani ER Bossi P Antunes HS Bensadoun R-J Systematic review of photobiomodulation for the management of oral mucositis in cancer patients and clinical practice guidelines. Supportive Care Cancer (2019) 27:3969–83. 10.1007/s00520-019-04890-2 31286228

[76] Gautam AP Fernandes DJ Vidyasagar MS Maiya AG Nigudgi S Effect of low-level laser therapy on patient reported measures of oral mucositis and quality of life in head and neck cancer patients receiving chemoradiotherapy-a randomized controlled trial. Supportive Care Cancer (2013) 21:1421–8. 10.1007/s00520-012-1684-4 23224689

[77] Alejandro Gonzalez-Arriagada W Alencar Ramos LM Carvalho Andrade MA Lopes MA Efficacy of low-level laser therapy as an auxiliary tool for management of acute side effects of head and neck radiotherapy. J Cosmetic Laser Ther (2018) 20:117–22. 10.1080/14764172.2017.1376097 29020483

[78] Marin-Conde F Castellanos-Cosano L Pachon-Ibanez J Serrera-Figallo MA Gutierrez-Perez JL Torres-Lagares D Photobiomodulation with low-level laser therapy reduces oral mucositis caused by head and neck radio-chemotherapy: prospective randomized controlled trial. Int J Oral Maxillofacial Surg (2019) 48:917–23. 10.1016/j.ijom.2018.12.006 30591391

[79] Alinca SB Saglam E Kandas NO Okcu O Yilmaz N Goncu B Comparison of the efficacy of low-level laser therapy and photodynamic therapy on oral mucositis in rats. Lasers Med Sci (2019) 34:1483–91. 10.1007/s10103-019-02757-w 30826950

[80] Jin T Li K-X Li P-J Huang S Chen X-Z Chen M An evaluation of nutrition intervention during radiation therapy in patients with locoregionally advanced nasopharyngeal carcinoma. Oncotarget (2017) 8:83723–33. 10.18632/oncotarget.19381 PMC5663549 29137377

[81] Kabarriti R Bontempo A Romano M McGovern KP Asaro A Viswanathan S The impact of dietary regimen compliance on outcomes for HNSCC patients treated with radiation therapy. Support Care Cancer (2018) 26:3307–13. 10.1007/s00520-018-4198-x 29671062

[82] Kang W-X Li W Huang S-G Dang Y Gao H Effects of nutritional intervention in head and neck cancer patients undergoing radiotherapy: A prospective randomized clinical trial. Mol Clin Oncol (2016) 5:279–82. 10.3892/mco.2016.943 PMC4998005 27588193

[83] Ravasco P Nutritional support in head and neck cancer: how and why? Anticancer Drugs (2011) 22:639–46. 10.1097/CAD.0b013e328345b4c5 21448060

[84] Corry J Poon W McPhee N Milner AD Cruickshank D Porceddu SV Randomized study of percutaneous endoscopic gastrostomy versus nasogastric tubes for enteral feeding in head and neck cancer patients treated with (chemo)radiation. J Med Imaging Radiat Oncol (2008) 52:503–10. 10.1111/j.1440-1673.2008.02003.x 19032398

[85] Alhambra Expósito MR Herrera-Martínez AD Manzano García G Espinosa Calvo M Bueno Serrano CM Gálvez Moreno M Early nutrition support therapy in patients with head-neck cancer. Nutricion hospitalaria (2018) 35:505–10. 10.20960/nh.1560 29974754

[86] Wei J Wu J Meng L Zhu B Wang H Xin Y Effects of early nutritional intervention on oral mucositis in patients with radiotherapy for head and neck cancer. QJM monthly J Assoc Physicians (2020) 113:37–42. 10.1093/qjmed/hcz222 31432089

[87] Wang C-H Wang H-M Pang Y-P Yeh K-Y Early nutritional support in non-metastatic stage IV oral cavity cancer patients undergoing adjuvant concurrent chemoradiotherapy: analysis of treatment tolerance and outcome in an area endemic for betel quid chewing. Supportive Care Cancer (2012) 20:1169–74. 10.1007/s00520-011-1192-y 21597937

[88] Kono M Wakisaka R Kumai T Hayashi R Komatsuda H Sato R Effects of early nutritional intervention by a nutritional support team for patients with head and neck cancer undergoing chemoradiotherapy or radiotherapy. Head Neck (2020) 43(2):514–19. 10.1002/hed.26502 33015926

[89] Meng L Wei J Ji R Wang B Xu X Xin Y Effect of Early Nutrition Intervention on Advanced Nasopharyngeal Carcinoma Patients Receiving Chemoradiotherapy. J Cancer (2019) 10:3650–6. 10.7150/jca.33475 PMC6636293 31333782

[90] Garabige V Giraud P De Rycke Y Girod A Jouffroy T Jaulerry C [Impact of nutrition management in patients with head and neck cancers treated with irradiation: is the nutritional intervention useful?]. Cancer Radiother (2007) 11:111–6. 10.1016/j.canrad.2006.11.005 17218137

[91] Ho YW Yeh KY Hsueh SW Hung CY Lu CH Tsang NM Impact of early nutrition counseling in head and neck cancer patients with normal nutritional status. Support Care Cancer (2020). 10.1007/s00520-020-05804-3 32995998

[92] Paccagnella A Morello M Da Mosto MC Baruffi C Marcon ML Gava A Early nutritional intervention improves treatment tolerance and outcomes in head and neck cancer patients undergoing concurrent chemoradiotherapy. Supportive Care Cancer (2010) 18:837–45. 10.1007/s00520-009-0717-0 19727846

[93] Sandmael JA Sand K Bye A Solheim TS Oldervoll L Helvik A-S Nutritional experiences in head and neck cancer patients. Eur J Cancer Care (2019) 28(6):e13168. 10.1111/ecc.13168 31571296

[94] Atasoy BM Yonal O Demirel B Dane F Yilmaz Y Kalayci C The impact of early percutaneous endoscopic gastrostomy placement on treatment completeness and nutritional status in locally advanced head and neck cancer patients receiving chemoradiotherapy. Eur Arch Oto Rhino Laryngol (2012) 269:275–82. 10.1007/s00405-010-1477-7 21472468

[95] Rutter CE Yovino S Taylor R Wolf J Cullen KJ Ord R Impact of Early Percutaneous Endoscopic Gastrostomy Tube Placement on Nutritional Status and Hospitalization in Patients With Head and Neck Cancer Receiving Definitive Chemoradiation Therapy. Head Neck (2011) 33:1441–7. 10.1002/hed.21624 21928416

[96] Piquet MA Ozsahin M Larpin I Zouhair A Coti P Monney M Early nutritional intervention in oropharyngeal cancer patients undergoing radiotherapy. Supportive Care In Cancer (2002) 10:502–4. 10.1007/s00520-002-0364-1 12353130

[97] González-Rodríguez M Villar-Taibo R Fernández-Pombo A Pazos-Couselo M Sifontes-Dubón MA Ferreiro-Fariña S Early versus conventional nutritional intervention in head and neck cancer patients before radiotherapy: benefits of a fast-track circuit. Eur J Clin Nutr (2020). 10.1038/s41430-020-00786-1 33097829

[98] Caccialanza R Cereda E Caraccia M Klersy C Nardi M Cappello S Early 7-day supplemental parenteral nutrition improves body composition and muscle strength in hypophagic cancer patients at nutritional risk. Support Care Cancer (2019) 27:2497–506. 10.1007/s00520-018-4527-0 30387050

[99] Isenring EA Capra S Bauer JD Nutrition intervention is beneficial in oncology outpatients receiving radiotherapy to the gastrointestinal or head and neck area. Br J Of Cancer (2004) 91:447–52. 10.1038/sj.bjc.6601962 PMC2409852 15226773

